# Genome Sequence of the Acidophilic Bacterium *Acidocella* sp. Strain MX-AZ02

**DOI:** 10.1128/genomeA.00041-12

**Published:** 2013-01-15

**Authors:** Luis E. Servín-Garcidueñas, Roger A. Garrett, Ricardo Amils, Esperanza Martínez-Romero

**Affiliations:** a Centro de Ciencias Genómicas, Universidad Nacional Autónoma de México, Cuernavaca, Morelos, Mexico; b Department of Biology, Archaea Centre, University of Copenhagen, Copenhagen, Denmark; c Centro de Biología Molecular Severo Ochoa, Universidad Autónoma de Madrid, Madrid, Spain

## Abstract

Here, we report the draft genome sequence of *Acidocella* sp. strain MX-AZ02, an acidophilic and heterotrophic alphaproteobacterium isolated from a geothermal lake in western Mexico.

## GENOME ANNOUNCEMENT

*Acidocella* sp. strain MX-AZ02 was isolated from a naturally acidic (pH 2.3) and heavy metal-containing shallow lake in the Los Azufres National Park in western Mexico. The *Acidocella* genus comprises aerobic, acidophilic, Gram-negative bacteria belonging to the class *Alphaproteobacteria* (1). *Acidocella* relatives have been identified both in natural and acid mine drainage environments exhibiting high heavy-metal levels (2–6). *Acidocella* has also been detected among *Sphagnum* moss microbiota growing under varying acidic conditions (7, 8). Currently, the genus contains three reference strains isolated from acidic environments (9–11).

DNA was isolated from *Acidocella* sp. MX-AZ02, which yields smooth, round, and translucent colonies on DSMZ medium 35a. The organism is maintained at the Center of Genomic Sciences in the culture collection of the Ecological Genomics Department, National University of Mexico (UNAM). The sequencing was performed with the Roche 454 GS-FLX titanium technology generating 58.04 Mbp (~16-fold coverage) from a mate-paired library with 3-kb inserts. The reads were assembled *de novo* using Newbler assembler 2.3 (454 Life Sciences). The assembly produced 303 contigs of >500 bp each with an N_50_ size of 22.12 kb. Nine scaffolds were generated containing 250 contigs. Genome annotation was performed using the NCBI Prokaryotic Genomes Automatic Annotation Pipeline (PGAAP) (http://www.ncbi.nlm.nih.gov/genomes/static/Pipeline.html).

The genome of *Acidocella* sp. MX-AZ02 was estimated to be 3.6 Mbp with a G+C content of 64.1% and it carried 3,553 open reading frames (ORFs). The 16S rRNA gene phylogeny indicated that the strain is closely related to type strains *Acidocella facilis* PW2, *Acidocella aluminiidurans* AL46, and *Acidocella aminolytica* 101, sharing 99.86%, 99.50%, and 97.93% sequence identities, respectively, over 1,407 bp.

Metal resistance determinants have been identified for *Acidocella* strains (12, 13, 14). The *Acidocella* sp. MX-AZ02 genome codes for arsenic, chromium, copper, and cobalt-zinc-cadmium transporters, as well as heavy-metal sensor signal transduction histidine kinases and chaperones. Carbonic anhydrases were also encoded, which may provide a means to cope with the low CO_2_ levels in acidic waters.

One *Acidocella* strain was shown to metabolize fructose from medium containing cell-free algal exudates, but it was unable to metabolize mannitol or glucose (15). *Acidocella* sp. MX-AZ02 may use glucose in the isolation medium as a carbon source. An acidophilic and abundant unicellular green alga was recently characterized from the same lake from which *Acidocella* sp. MX-AZ02 was isolated (16). Possibly, *Acidocella* sp. MX-AZ02 utilizes organic compounds from the alga, as was proposed previously for acidophilic microalgae and acidophilic heterotrophic bacteria (15).

The draft genome of *Acidocella* sp. MX-AZ02 will facilitate the identification of metal resistance determinants and may help us understand bacterial–algal interactions. This is the first isolated bacterial genome for an *Acidocella* strain and is the first sequenced bacterial genome from the Los Azufres National Park.

### Nucleotide sequence accession number. 

The draft of the genome sequence is deposited at DDBJ/EMBL/GenBank under the accession no. AMPS00000000.
